# The multi-omics analyses of acsl1 reveal its translational significance as a tumor microenvironmental and prognostic biomarker in clear cell renal cell carcinoma

**DOI:** 10.1186/s13000-023-01384-y

**Published:** 2023-08-22

**Authors:** Yang Yang, Jiayu Liang, Junjie Zhao, Xinyuan Wang, Dechao Feng, Hang Xu, Yu Shen, Yaowen Zhang, Jindong Dai, Zhipeng Wang, Qiang Wei, Zhenhua Liu

**Affiliations:** 1 Department of Urology, Institute of Urology, West China Hospital, Sichuan University, Chengdu, 610041 China; 2 Department of Urology, The First People’s Hospital of Jiujiang in Jiangxi Province, No. 48, Taling South Road, Xunyang District, Jiujiang City, 332000 Jiangxi Province China; 3 No.37 Guoxue Alley, Wuhou District, Chengdu City, Sichuan Province PR China

**Keywords:** ACSL1, Clear cell renal cell carcinoma, Methylation, Immune Microenvironment, m6A modification

## Abstract

**Background:**

Clear cell renal cell carcinoma (ccRCC) is the dominant subtype of kidney cancer. Dysregulation of long-chain acyl-CoA synthetase 1 (ACSL1) is strongly implicated in undesirable results in varieties of cancers. Nevertheless, the dysregulation and associated multi-omics characteristics of ACSL1 in ccRCC remain elusive.

**Methods:**

We probed the mRNA and protein profiles of ACSL1 in RCC using data from the Cancer Genome Atlas, Gene Expression Omnibus, the Human Protein Atlas (HPA), and Clinical Proteomic Tumor Analysis Consortium (CPTAC) and verified them in our patient cohort and RCC cell lines. Correlations between ACSL1 expression and clinicopathological features, epigenetic modification and immune microenvironment characteristics were analyzed to reveal the multi-omics profile associated with ACSL1.

**Results:**

ACSL1 was down-regulated in ccRCC tissues compared to adjacent normal tissues. Lower expression of ACSL1 was linked to unfavorable pathological parameters and prognosis. The dysregulation of ACSL1 was greatly ascribed to CpG island-associated methylation modification. The ACSL1 high-expression subgroup had enriched fatty acid metabolism-related pathways and high expression of ferroptosis-related genes. In contrast, the ACSL1 low-expression subgroup exhibited higher immune and microenvironment scores, elevated expression of immune checkpoints PDCD1, CTLA4, LAG3, and TIGIT, and higher TIDE scores. Using data from the GDSC database, we corroborated that down-regulation of ACSL1 was associated with higher sensitivity towards Erlotinib, Pazopanib, and PI3K-Akt-mTOR-targeted therapeutic strategies.

**Conclusion:**

Taken together, our findings point to ACSL1 as a biomarker for prognostic prediction of ccRCC, identifying the tumor microenvironment (TME) phenotype, and even contributing to treatment decision-making in ccRCC patients.

**Supplementary Information:**

The online version contains supplementary material available at 10.1186/s13000-023-01384-y.

## Introduction

Renal cell carcinoma (RCC), the most prevalent form of kidney cancer, arises from renal tubular epithelial cells and comprises more than 80% of adult renal malignancies [1]. RCC is the sixth and the ninth most frequently occurring malignancy in men and women, respectively. Estimates suggest that there will be around 79,000 new cases of RCC in the US in 2022 [1]. Clear cell renal cell carcinoma (ccRCC) is the most commonly occurring subtype of RCC, making up about 75% of all cases [2]. Surgical procedures can serve as an effective therapeutic procedure for ccRCC during localized or locally advanced stages. Systemic treatment provides relief but no cure for patients with advanced diseases [3]. Over the past decade, RCC has been regarded as a metabolic disease [3, 4]. For example, RCC retains the ability to rewire the normal metabolism to cope with oxygen deprivation despite the inactivation of Von Hippel Lindau (VHL). Although under the normoxia, ccRCC produces the aberrant transcription factors hypoxia-inducible factor (HIF)1α and HIF2α [5, 6], which in turn stimulate the pathways involved in fatty acid, glycolysis, and glycogen synthesis [7–9]. Moreover, a recent study suggested that targeting metabolic pathways in kidney cancer would be the potential therapy [10].

Long-chain acyl-CoA synthetase 1 (ACSL1), which can activate the long-chain fatty acids (LCFA; 12–20 carbons) to fatty acyl-CoAs by esterification, is a member of the ACSLs family [11, 12]. The lipid metabolism network can play various kinds of metabolic roles, including energy production, temperature regulation, and molecular signal synthesis[13, 14]. Dysregulation of fatty acid metabolism gives rise to an overload of lipid biosynthesis and deposition, which ultimately leads to the development of metabolic diseases, cardiovascular diseases and cancer[15]. Various sets of papers have demonstrated the connections between ACSL1 and multiple cancers. For instance, ACSL1 is up-regulated in colorectal cancer and estrogen receptor (ER)-negative, ER-positive and HER2-positive breast cancer subtypes, and high ACSL1 expression in these patients’ tumor samples is linked to unfavorable prognosis [12, 16–19]. In parallel, ACSL1 has been previously reported to be down-regulated in non-small cell lung cancer (NSCLC) and liver cancer, with its tumor-suppressive effects in NSCLC having been demonstrated by Chen, W.C et al. [19, 20]. The differential ACSL1 expression in different tumors suggests that ACSL1 has diverse effects on tumorigenesis, which is worth studying. Nevertheless, the expression characteristics and potential mechanisms of ACSL1 in ccRCC tumorigenesis are still unclear.

The regulatory mechanism of aberrant ACSL1 expression has been reported in several studies. Wang et al. suggested that ACSL1 down-regulation was caused by copy number deletion in breast cancer [21]. Nevertheless, in the brown adipose tissue, DNA hypermethylation led to the decreased expression of ACSL1[22]. The role of dysregulation of the epigenome, like DNA/RNA methylation and histone modification, in driving cancer evolution and advancement, has been increasingly understood and highlighted. Given the fact that chromatin modifiers genes, including PBRM1, SETD2, and BAP1, were widely mutated, epigenetic modification also plays a crucial part in the abnormal modulation of genes resulting in ccRCC. Additionally, down-regulation of ACSL1 in breast cancer was associated with alteration of the mTOR signaling pathway, which might be a possible candidate for treatment, and the findings were consistent with another study conducted by Liśkiewicz et al. [23]. Nevertheless, as we know, the potential epigenetic modification of ACSL1 and its relationship with the mTOR signaling in ccRCC has not been researched yet.

Here, we addressed the differential expression of ACSL1 in ccRCC and its association with the prognosis. We identified that ACSL1 could be epigenetically regulated, with further findings that the ACSL1 expression was implicated in the immune microenvironment, ferroptosis-associated genes and the treatment strategies in ccRCC. These findings shed new light not only on the understanding of the function of ACSL1 in the advancement of ccRCC, but also on the finding that ACSL1 expression can suggest different multi-omic molecular typing and promising therapeutic approaches in ccRCC.

## Materials and methods

### Cell culture and samples

The ccRCC cell lines with the inclusion of 786-O, 769-P and OS-RC2 were cultured using RPMI-1640 (Invitrogen), and HK-2 (renal tubular epithelial cells) were kept in the Dulbecco’s modified Eagle’s medium (DMEM, Invitrogen), with all medium containing 10% fetal bovine serum (FBS, Hyclone). ccRCC cell lines and HK-2 were acquired from the American Tissue Culture Collection (ATCC). The ccRCC tissues (n = 29) and paracancerous tissues (n = 29) were obtained from our ccRCC tissues biobank. Each participant subscribed to a prior informed consent form. The Ethics Committee of West China Hospital, Sichuan University (Chengdu, China) authorized the protocol for this research.

### Expression of ACSL1 in ccRCC

We used TCGA data sets (www.tcga-data.nci.nih.gov/tcga) [24] to investigate differential expression of ACSL1 in multiple cancers and displayed the data using a box plot via Tumor Immune Estimation Resource (TIMER) [25, 26]. In our study, 72 normal tissues and 533 tumor samples for ccRCC were subjected to analysis of ACSL1 RNA expression patterns. The TCGA dataset was employed to verify the expression profiles of ACSL1 in cancerous samples and controls using the Wilcoxon test. Gene expression is represented as log2 TPM values. We also adopted the Wilcoxon rank sum test to investigate the GEO (www.ncbi.nlm.nih.gov/geo; GSE53757, n = 144 and GSE40435, n = 202) [13] data sets to probe the differentiated ACSL1 RNA expression between ccRCC and control groups.

### Quantitative real-time PCR (qRT- PCR)

Total RNA was derived out of tissues and cells with the aid of the RNeasy Total RNA Isolation Kit (Qiagen) and then reversely transcribed by utilizing the Quantitative Reverse Transcription Kit (QIAGEN). qRT-PCR was conducted with an A25742SYBR™ Green Master Mix. The data were standardized using GAPDH as endogenous control and further analyzed with the 2^−ΔΔCT^ method. The primers (5′ to 3′) used are as follows: ACSL1 forward: 5’-GACATTGGAAAATGGTTACCAAATG − 3’ and reverse: 5’-GGCTCACTTCGCATGTAGATA − 3’. GAPDH forward: 5’-GGAGCGAGATCCCTCCAAAAT-3’ and reverse: 5’GGCTGTTGTCATACTTCTCATGG-3’.

### Protein expression of ACSL1 in ccRCC

The proteins from tissues and cells were extracted by lysing with RIPA comprising 1% protease inhibitors. The BCA assay was conducted to determine the protein content. Western blotting was conducted with reference to previous studies [27–29]. The following primary antibodies and dilutions were applied: the ACSL1 antibody (proteintech, 13989-1-AP) (diluted at 1:3000) and the GAPDH recombinant antibody (proteintech, 80570-1-RR) (diluted at 1:1000).

Immunohistochemical images of ACSL1 protein in cancerous tissues from ccRCC patients (ID: 3541) as well as associated normal kidney tissues (ID: 1859) were from the Human Protein Atlas (HPA) (http://www.proteinatlas.org/) [29–31]. Atlas Antibodies Sigma-Aldrich provided the HPA011316 antibody (0.0475 mg/mL) for immunohistochemistry (IHC). Next, we used Clinical Proteomic Tumor Analysis Consortium (CPTAC) to study the differentiation in ACSL1 expression between cancerous and control tissues by the UALCAN tool [32].

### Clinicopathology and prognosis analysis

The interaction between ACSL1 expression and clinicopathological parameters (age, gender, T-stage, N-stage, M-stage, pathological stage, histological grading, etc.) in the TCGA-ccRCC cohort was integrated and examined. The measurement data were presented as the mean ± SD. An unpaired t-test was employed for statistical assessments. Pearson chi-squared test or Fisher’s exact test was made available to probe the link between ACSL1 and characteristic clinical variances [33]. The prognostic and diagnostic potential of ACSL1 in ccRCC was assessed by overall survival (OS), disease-specific survival (DSS) and progression-free survival (PFS) analysis, which were exhibited by Kaplan-Meier curves and survival charts, with data from the TCGA database [34, 35]. The prognostic effect of the model could be gauged by the receiver operating characteristic (ROC) curve analysis [36]. Hence, ROC analyses were created by applying the R package “pROC” to study the area beneath the ROC curve (AUC) to assess the sensitivity (true positive rate) and specificity (true negative rate) of ACSL1 for ccRCC diagnosis.

### DNA methylation analysis

We adopted the R software package “ggplot2” to figure out the connection between DNA methylation-related genes with high and low expression of ACSL1 in ccRCC tissues. The DNA methylation-related genes including TRDMT1, TET1, TET2, TET3, DNMT1, DNMT3A, DNMT3B, DNMT3L [37], then we resorted to Spearman rank test to determine the correlation between the ACSL1 expression and the DNA methylation-related genes and visualized by heatmap. By using the SMART tool [38], the methylation extent of ACSL1 in normal and tumor samples was compared, and the definite correspondence between the methylation degree and the ACSL1 expression in ccRCC was gauged. We obtained Beta values for analysis and the Pearson method was performed to measure the association between methylation degrees and ACSL1 expression. Then, we resorted to the MethSurv tool (https://biit.cs.ut.ee/methsurv/) to study the methylation status of diversified probes, according to the datasets of the TCGA-ccRCC database, and visualized the results with the waterfall plot [39]. We then turned our attention to highly methylated probes to interrogate the underlying association of these probes with prognosis. The outcomes were shown by the Kaplan–Meir plot.

### Relationships between ACSL1 expression and m6A modification and ferroptosis in the ccRCC cohort

We applied the R software package “ggplot2” to study the correspondence between the ACSL1 gene and the profiles of m6A-associated or ferroptosis-related genes in TCGA-ccRCC data sets. The m6A-related genes comprised ZC3H13, YTHDF3, HNRNPA2B1, IGF2BP1, IGF2BP3, YTHDC2, YTHDF1, FTO, HNRNPC, METTL14, METTL3, WTAP, RBM15, ALKBH5, IGF2BP2, RBMX, RBM15B, YTHDC1, VIRMA and YTHDF2 [40, 41]. In parallel, ferroptosis-related genes included LPCAT3, HSPA5, CARS1, CDKN1A, CS, GLS2, ALOX15, SAT1, ACSL4, EMC2, RPL8, FANCD2, NFE2L2, DPP4, TFRC, ATP5MC3, GPX4, FDFT1, MT1G, NCOA4, SLC7A11, HSPB1, CISD1, and SLC1A5 [42]. we firstly profiled the expression of m6A-related genes in the ACSL1 high- and low-expression group,then we investigated the expression of ferroptosis-related genes in ACSL1 high-expression group of ccRCC, ACSL1 low-expression group of ccRCC and adjacent normal kidney tissue from the TCGA dataset. Next, we separately screened out six m6A-related genes and ten ferroptosis-related genes with the lowest *P* value in the ACSL1 high- and low-expression group. At last, we probed whether the effects of these genes on prognosis were different between the two groups by univariate cox regression analysis [41–44].

### Enrichment assays of differentially expressed genes between ACSL1-high and -low-expression subgroups in ccRCC

The Limma package (version: 3.40.2) was utilized to characterize the varied expression of mRNAs between the ACSL1 high- and low-expression groups. (cut-off: median expression value) by analyzing the TCGA ccRCC data sets. Adjusted *P* < 0.05 and log (fold change) > 1.5 or <-1.5 was defined as the threshold for screening out differentially expressed mRNAs. A volcano plot was outlined to shed light on all the up-regulated and down-regulated mRNAs with statistical significance. The Kyoto Encyclopedia of Genes and Genomes (KEGG) pathway and the Gene ontology (GO) analysis of co-expressed genes was performed with the clusterProfiler package (version: 3.18.0) [45] of R software. Where enrichment results were available, *P* < 0.05 or FDR < 0.05 signified statistical significance.

### Immune infiltration and immune checkpoint analysis

To study the underlying relevance between ACSL1 expression to immunological conditions in the tumor microenvironment (TME) in ccRCC, we resorted to “immunedeconv” package to investigate the difference in the distribution of immune cell fractions in the ACSL1 high-expression group of ccRCC, the ACSL1 low-expression group of ccRCC and adjacent normal kidney tissue group from the TCGA dataset. xCELL algorithm was selected for analysis. A heatmap was plotted and the Kruskal-Wallis was exploited to establish the significance of these groups [46]. Meanwhile, we picked SIGLEC15, TIGIT, CD274, HAVCR2, PDCD1, CTLA4, LAG3 and PDCD1LG2 as immune checkpoint-associated transcripts [47–49]. These eight genes were exploited using the ‘ggplot2’ package to extract expression values in the ACSL1 high-expression group of ccRCC, the ACSL1 low-expression group of ccRCC and adjacent normal kidney tissue group from the TCGA dataset.These groups of samples were identified by the Kruskal-Wallis for significance. Next, corresponding clinical information was acquired from the TCGA-ccRCC cohort for the RNA sequencing data. We then predicted underlying immune checkpoint blockade (ICB) responses in the ACSL1 high- and low-expression group by adopting the Tumor Immune Dysfunction and Rejection (TIDE) algorithm. TIDE is a methodology for modeling the two principal mechanisms of tumor immune evasion, where high TIDE scores are related to inferior efficacy and reduced survival time following ICB treatment [51]. The findings were exhibited with the packages “ggplot2” and “ggpubr”.

### Potential therapeutic strategies analysis

We made predictions of chemotherapy response for ccRCC samples as per the Genomics of Drug Sensitivity in Cancer (GDSC) [50, 51]. TKI drugs (Sunitinib and Pazopanib) and the PI3K pathway inhibitors, including Temsirolimus and Pictilisib (GDC0941) were adopted. These drugs were employed to evaluate the therapeutic response of ACSL1 in the high-expression group and the ACSL1 low-expression group via half-maximal inhibitory concentration (IC50). The IC50 for each sample was assessed using ridge regression. All settings were configured by default and the removal of batch effects of combat and tissue type, and the duplicate gene expression was aggregated to the mean.

### Statistical analysis

R (v 4.0.3) software applied for statistical analysis together with the packages ggplot2, heatmap, ggpubr, pROC, Limma, survival, survminer, clusterProfiler and immunedeconv was applied to analyze the data. Wilcox test or T-test was used to analyse the statistical difference of two groups, Kruskal-Wallis one-way ANOVA was applied to analyse significance difference of three groups. Survival rates were assessed using Kaplan–Meier curves and log-rank tests.The correlation between two variables was used by Spearman rank test or Pearson’s test. A *P* value < 0.05 was deemed statistically valid.

## Results

### Dysregulation of ACSL1 in RCC tumor tissues

**Fig. 1:**
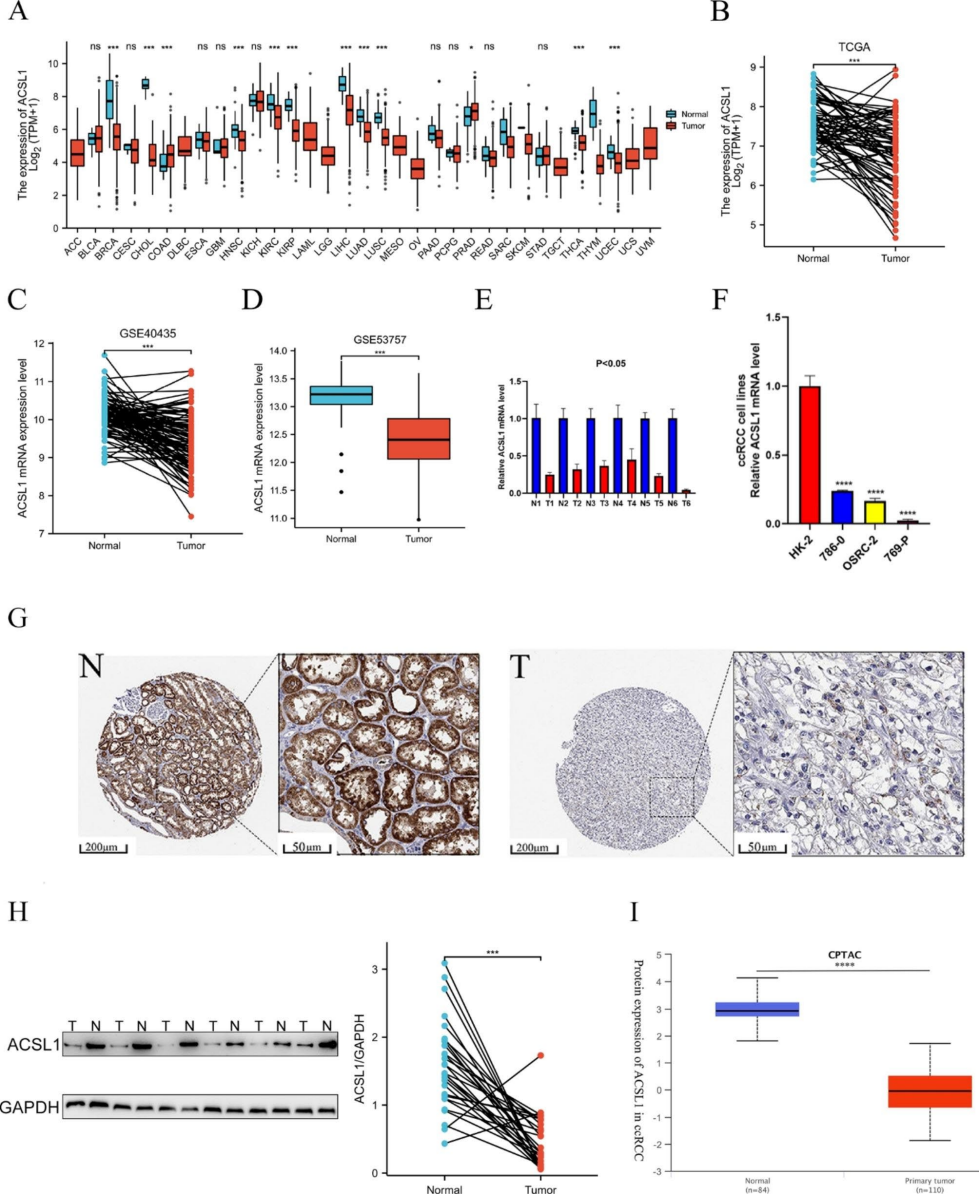
ACSL1 expression in RCC tissues and pan-carcinoma. **(A)** ACSL1 profiles in tumorous and normal tissues of pan-cancer data from the Cancer Genome Atlas (TCGA). **(B)** ACSL1 mRNA expression between ccRCC and matched healthy tissues in TCGA data sets. **(C)** ACSL1 mRNA expression in ccRCC and matched normal tissues in GSE40435 data sets. **(D)** Differential expression of ACSL1 between ccRCC and normal tissues in GSE53757 data. **(E)** ACSL1 mRNA expression levels in the six RCC and matched paracancerous tissues. **(F)** Differential expression of ACSL1 in ccRCC cell lines and renal tubular epithelial cell HK-2. **(G)** Immunohistochemical images from the HPA data sets show ACSL1 expression in RCC tissues and normal renal tissues. **(H)** Protein expression of ACSL1 in ccRCC tissues and neighboring healthy tissues from six patients in West China Hospital. N1-6: paracancerous tissues of patients 1–6, T1-6: cancer tissues of patients 1–6. the bands gray value of N1-6 significantly higher than the T1-6. The normalized gray value of 29 pairs of renal carcinoma tissues after quantitative analysis were shown on the right. **(I)** Protein expression of ACSL1 in tumor and normal tissues in ccRCC data from CPTAC. *P < 0.05; **P < 0.01; ***P < 0.001; ****P < 0.0001. ns, not significant

In the TCGA database, pan-cancer analysis exhibited that the ACSL1 displayed differential expression with statistical significance in multiple cancers, like Kidney renal clear cell carcinoma (KIRC) and Kidney renal papillary cell carcinoma (KIRP) (Fig. 1A). Besides, the expression of ACSL1 was significantly decreased in the paired ccRCC samples from the TCGA database (Fig. 1B, P < 0.001) and other two GEO datasets (Fig. 1C, D and P < 0.001). Through qRT-PCR experiments (Fig. 1E), we validated that the ACSL1 mRNA expression was distinctly lower in ccRCC tissues versus matched adjacent normal tissues from West China Hospital (Chengdu, Sichuan, China). Also, we observed that the ACSL1 mRNA expression was lower in ccRCC cell lines (786-O, OS-RC-2 and 769-P) than that in HK-2 (human kidney epithelial tubular cell line) (Fig. 1F).

To validate the results from the mRNA expression level, we next explored the ACSL1 protein expression through HPA. As a result, ACSL1 was mediumly expressed in the membrane and cytoplasm of cells in renal tubules with moderate intensity at a rate of 75%-25%, but it was barely detectable in ccRCC tumor cells (Fig. 1G). To provide further evidence of the accuracy of our observations, we performed Western Blot and uncovered that the protein expression of ACSL1 was almost entirely lower in ccRCC tumorous tissues versus adjacent normal tissues (Fig. 1H). Supplementary Figure S1 shows the result of western blot about the remaining 23 pairs of renal carcinoma tissues. At last, we further checked the data from CPTAC to validate the differences in protein expression of ACSL1 between tumorous and control tissues. As depicted in Fig. 1I, the protein expression of ACSL1 was markedly less in tumor tissues versus the corresponding normal samples. (*P* < 0.001).

### Down-regulation of ACSL1 was related to clinical stage and pathological grade as well as poor prognosis

**Fig. 2:**
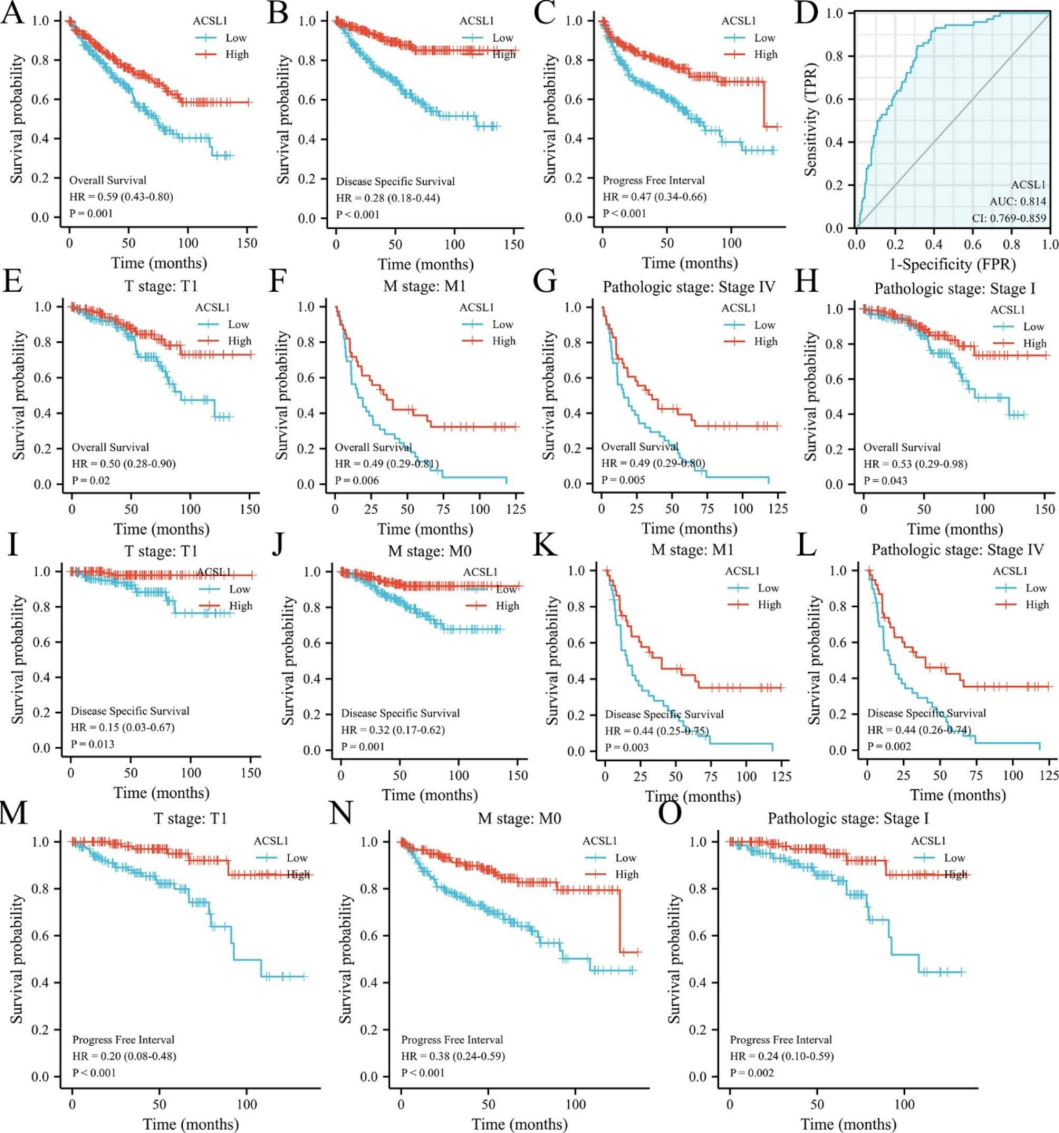
Clinical values and subgroup assessment of ACSL1 expression and survival in ccRCC. **(A)** The link between ACSL1 expression and OS in ccRCC. **(B)** The relationship between ACSL1 expression and DSS in ccRCC. **(C)** The association between ACSL1 expression and PFS mark. **(D)** Analysis of recipient operating characteristic profiles for ACSL1 diagnoses in ccRCC. **(E-H)** The correlation between ACSL1 expression and OS in stage T1, stage M1, pathology I and IV ccRCC. **(I-L)** The connection between ACSL1 expression and DSS in T1 stage, M0, M1 stage, and pathologic IV. **(M-O)** The link between ACSL1 expression and PFS in T1 stage, M1 stage and pathologic stage I

To further assess the prognostic and clinicopathological significance of ACSL1 expression in ccRCC, we used the TCGA-KIRC data cohort to stratify patients into the high-or low-expression group with regard to median ACSL1 expression. The baseline characters and therapeutic outcomes within different ACSL1 expression groups are depicted in Table 1. Kaplan-Meier curves and log-rank tests displayed that low expression of ACSL1 in ccRCC corroborated with shortened OS (*P* = 0.001, Fig. 2A), DFS (*P* < 0,001, Fig. 2B) and PFS (*P* < 0.001, Fig. 2C). The ROC curve applied to assess the sensitivity and specificity of ACSL1 for the outcome prediction of ccRCC in TCGA data sets, exhibiting an area beneath the ROC curve of 0.814 (95% CI: 0.769–0.859) (Fig. 2D). For patients in T1 stage, M1 stage, pathologic stage I and IV were significantly associated with worse OS (Fig. 2E-H, P < 0.05), For patients in T1 stage, M0 and M1 stage, pathologic stage IV were significantly related to poorer DFS (Fig. 2I-L, P < 0.05), For patients in T1 stage, M0 stage, pathologic stage I were implicated in shorter PFS (Fig. 2M-O, P < 0.05), All these findings illustrated that lower expression levels of ACSL1 have coincided with higher clinical stage, pathological grade, and poorer prognosis.

**Table 1:** Relationships between clinical features and ACSL1 expression in ccRCC patients in TCGA data sets

Characteristic	Low expression of ACSL1	High expression of ACSL1	p
n	269	270	
T stage, n (%)			0.013
T1	121 (22.4%)	157 (29.1%)	
T2	38 (7.1%)	33 (6.1%)	
T3	102 (18.9%)	77 (14.3%)	
T4	8 (1.5%)	3 (0.6%)	
N stage, n (%)			0.068
N0	116 (45.1%)	125 (48.6%)	
N1	12 (4.7%)	4 (1.6%)	
M stage, n (%)			0.011
M0	204 (40.3%)	224 (44.3%)	
M1	50 (9.9%)	28 (5.5%)	
Pathologic stage, n (%)			0.002
Stage I	115 (21.5%)	157 (29.3%)	
Stage II	30 (5.6%)	29 (5.4%)	
Stage III	69 (12.9%)	54 (10.1%)	
Stage IV	53 (9.9%)	29 (5.4%)	
Primary therapy outcome, n (%)			0.295
PD	7 (4.8%)	4 (2.7%)	
SD	2 (1.4%)	4 (2.7%)	
PR	0 (0%)	2 (1.4%)	
CR	51 (34.7%)	77 (52.4%)	
Gender, n (%)			< 0.001
Female	69 (12.8%)	117 (21.7%)	
Male	200 (37.1%)	153 (28.4%)	
Race, n (%)			0.432
Asian	6 (1.1%)	2 (0.4%)	
Black or African American	29 (5.5%)	28 (5.3%)	
White	232 (43.6%)	235 (44.2%)	
Age, n (%)			0.282
<=60	141 (26.2%)	128 (23.7%)	
> 60	128 (23.7%)	142 (26.3%)	
Histologic grade, n (%)			< 0.001
G1	5 (0.9%)	9 (1.7%)	
G2	96 (18.1%)	139 (26.2%)	
G3	110 (20.7%)	97 (18.3%)	
G4	54 (10.2%)	21 (4%)	
Laterality, n (%)			1.000
Left	126 (23.4%)	126 (23.4%)	
Right	142 (26.4%)	144 (26.8%)	
OS event, n (%)			< 0.001
Alive	156 (28.9%)	210 (39%)	
Dead	113 (21%)	60 (11.1%)	
DSS event, n (%)			< 0.001
Alive	180 (34.1%)	240 (45.5%)	
Dead	83 (15.7%)	25 (4.7%)	
PFI event, n (%)			< 0.001
Alive	158 (29.3%)	220 (40.8%)	
Dead	111 (20.6%)	50 (9.3%)	
Serum calcium, n (%)			0.880
Elevated	6 (1.6%)	4 (1.1%)	
Low	103 (28.1%)	100 (27.3%)	
Normal	79 (21.6%)	74 (20.2%)	
Hemoglobin, n (%)			0.120
Elevated	3 (0.7%)	2 (0.4%)	
Low	147 (32%)	116 (25.3%)	
Normal	88 (19.2%)	103 (22.4%)	
Age, mean ± SD	60.04 ± 12.32	61.21 ± 11.86	0.259

### DNA methylation serves as a promising down-regulatory mechanism of ACSL1 expression in ccRCC

**Fig. 3:**
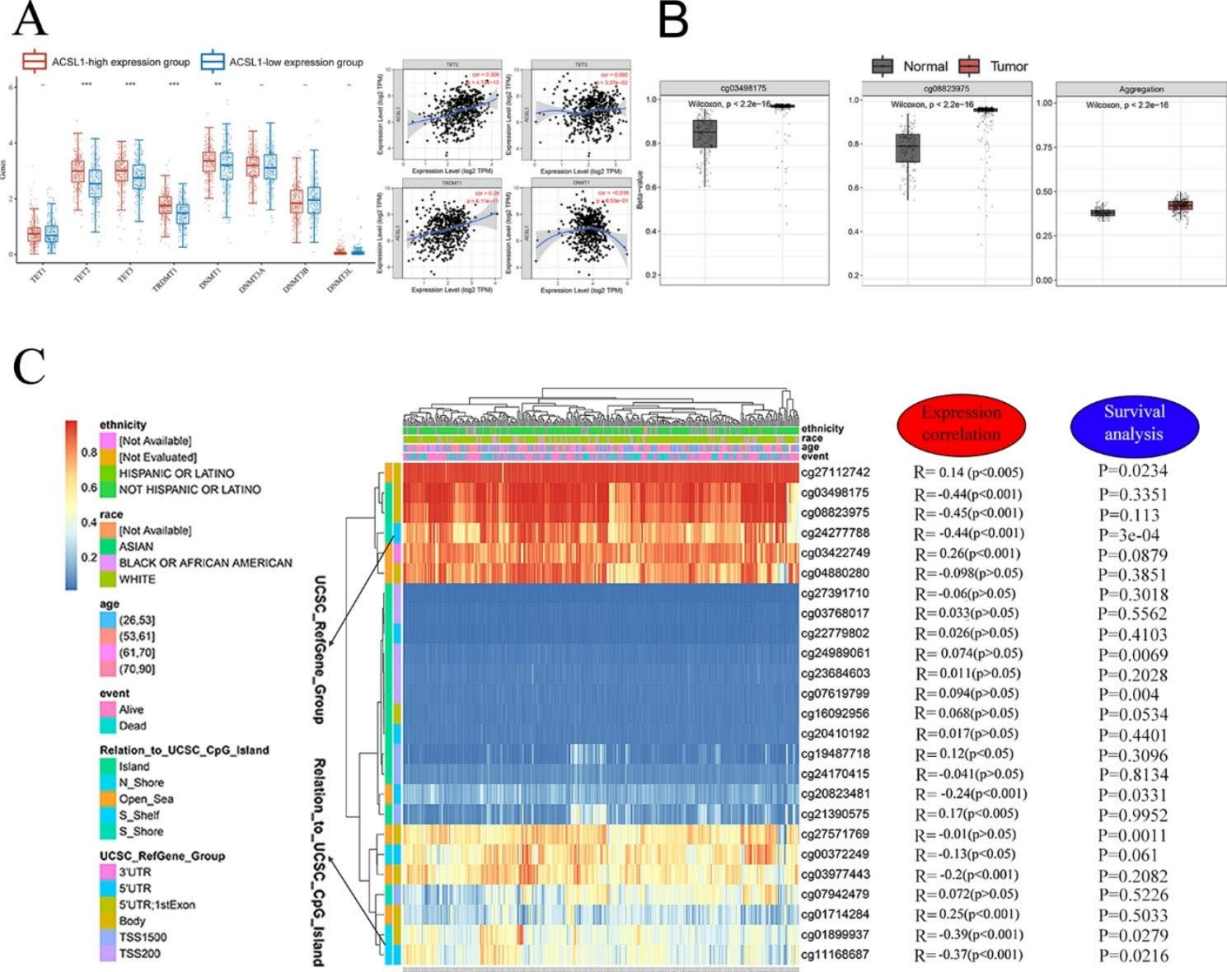
DNA methylation modification was associated with ACSL1 silence. (A) Changes of 8 DNA methylation-associated genes between ACSL1-low and ACSL1-high expression subgroups in ccRCC. Correlation of ACSL1 mRNA expression with TET2, TET3, TRDMT1 on the right. *P < 0.05; **P < 0.01; ***P < 0.001; -, not significant. (B) Comparison of DNA methylation probes between healthy and tumorous samples: cg03498175 (ACSL1) and cg08823975 (ACSL1) exhibited higher methylation levels in tumor tissues, *P* < 0.0001. (C) A waterfall plot of the methylation level of the ACSL1 subregions in ccRCC is presented. The association between methylation levels of different ACSL1 probes with expression and survival were analyzed

Next, we sought to elucidate the potential mechanism for the down-regulation of ACSL1 in ccRCC. Firstly, we found that tumors with higher ACSL1 expression had significantly higher expression of DNA methylation-associated genes TET2, TET3, TRDMT1 and DNMT1. Moreover ACSL1 gene was positively correlated with these DNA methylation-associated genes TET2 (R = 0.306, P = 4.72e-13), TET3 (R = 0.092, P = 3.37e-2), TRDMT1(R = 0.28, P = 6.11e-11), DNMT1(R=-0,019, P = 6.53e-01)Fig. 3A, P < 0.05). These evidences suggesting that the transcriptomic regulation of ACSL1 may be linked to DNA methylation status. We then explored the differential DNA methylation levels of ACSL1 between tumor and normal tissues, discovering that the aggerated methylation for all the probes in ACSL1 gene loci was more pronounced in tumor tissues versus normal tissues. Specifically, the GpG island-related probes cg03498175 and cg08823975 exhibited remarkably higher methylation levels in tumor tissues (Fig. 3B, Supplementary Figure S2, *P* < 0.001).

We then depicted a heatmap about the connection between methylation levels, gene subregions and gene expression in light of the sequencing results in the TCGA-ccRCC cohort. The outcomes disclosed that 8 out of 14 methylation sites in ccRCC had obviously negative correlation with ACSL1 expression, including cg03498175(r=-0.44, *P* < 0.001), cg08823975(r=-0.45, *P* < 0.001), cg24277788(r=-0.44, *P* < 0.001), cg01899937(r=-0.39, *P* < 0.001) and cg111668687(r=-0.37, *P* < 0.001) (Fig. 3C). Supplementary Figure S3 shows detailed correlations between the ACSL1 expression with the methylation sites. Moreover, we analyzed the prognostic significance of these ACSL1-associated methylation loci and revealed that patients with a greater extent of methylation of cg20823481, cg24277788, cg01899937 and cg111668687 had worse OS (Fig. 3C, P < 0.05). Overall, these findings illustrated that ACSL1-related DNA methylation modification was strongly linked to its ACSL1 expression and ccRCC patients’ prognosis.

### The association between ACSL1 with m6A modification-related genes in ccRCC

**Fig. 4:**
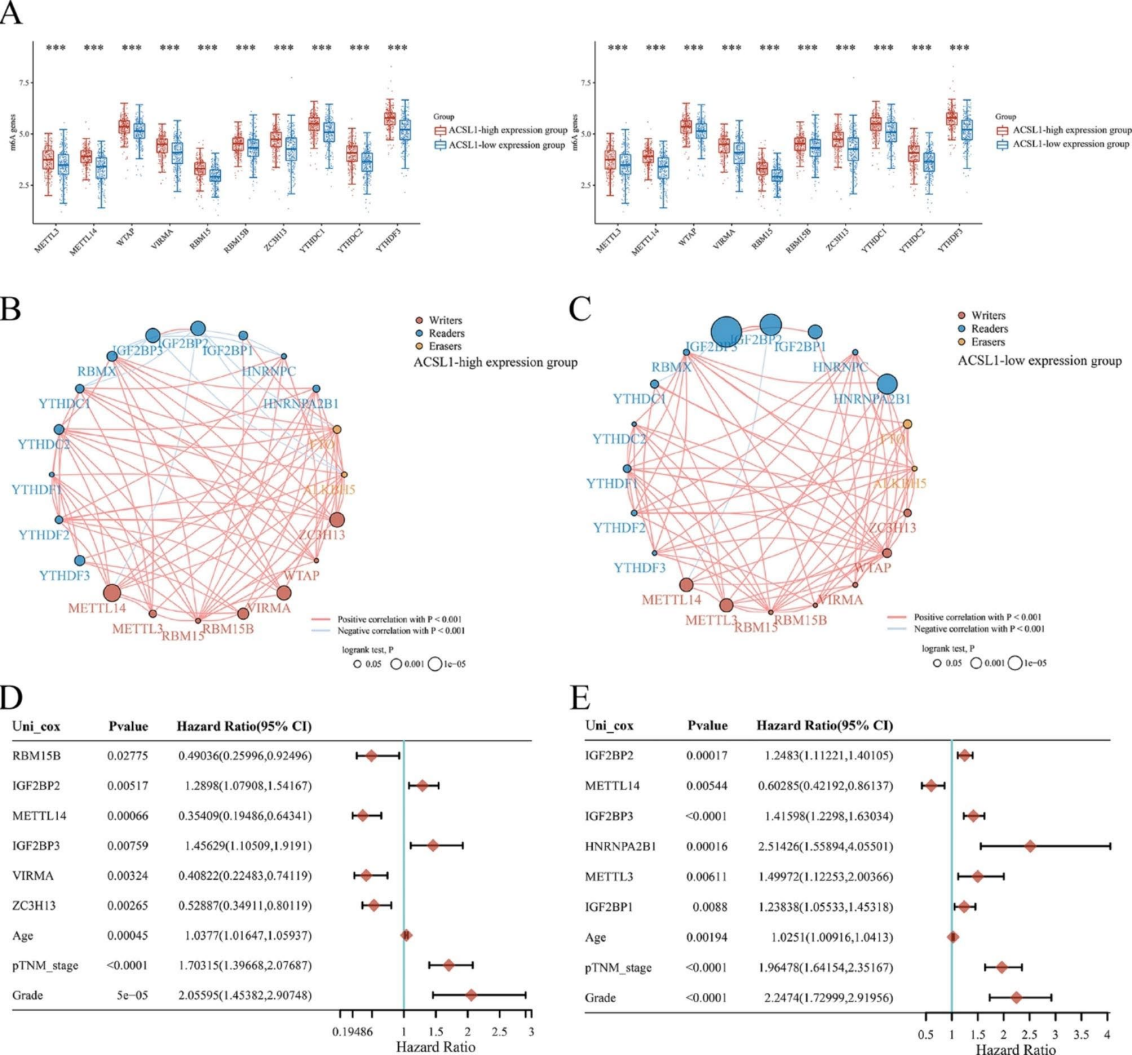
The relationship of ACSL1 expression with m6A-related genes in ccRCC. **(A)** The differentially expressed m6A-associated genes between the ACSL1 high- and low-expression group in ccRCC. *P < 0.05, **P < 0.01, ***P < 0.001. **(B-C)** The connection between m6A-associated genes in ACSL1 high- and low-expression groups. **(D-E)** Univariate Cox regression analysis of age, clinical stage, tumor grade, and six m6A-associated genes with the smallest *P* value in predicting prognosis in the ACSL1 high-expression group (D) and the ACSL1 low-expression group (E) in ccRCC

m6A modification has a critical function in ccRCC[40]. The correspondence between the expression of ACSL1 and that of m6A-related genes was researched by analyzing TCGA- ccRCC samples. The results demonstrated that the ACSL1 expression exhibited a notable correlation with the expression of m6A writers (ZC3H13, WTAP, VIRMA, RBM15, METTL3, and METTL14), m6A readers (YTHDF1, YTHDF2, YTHDF3, YTHDC1, YTHDC2, RBMX, HNRNPC, and HNRNPA2B1), and m6A erasers (FTO and ALKBH5) (Fig. 4A, P < 0.05) in TCGA ccRCC data sets. Additionally, the ACSL1 expression was significantly and reversely associated with the expressions of m6A readers (IGF2BP2 and IGF2BP3) (Fig. 4A, P < 0.05). Next, we investigated the correlations among the m6A-related genes in ACSL1 high- and low-expression groups, respectively. (Fig. 4B-C). Then we selected a total of six genes with the smallest *P* value in predicting prognosis in ACSL1 high-expression subgroups, including m6A writers (METTL14, ZC3H13, VIRMA and RBM15B) and m6A readers (IGF2BP2 and IGF2BP3). Within the ACSL1 low-expression group, six genes had minimal *P* values, including m6A reader (IGF2BP1, IGF2BP2, IGF2BP3 and HNRNPA2B1) and m6A writers (METTL14 and METTL3). Subsequently, we explored the prognostic merits of these m6A-associated genes in ACSL1 high-expression subgroups and ACSL1 low-expression subgroups, respectively (Fig. 4D-E). These results suggested that m6A-related genes had different prognostic values in ACSL1-high subgroups and ACSL1-low subgroups of ccRCC, and these prognostic correlations might be influenced by the expression levels of ACSL1 in ccRCC patients.

### Enrichment analysis of ACSL1-associated functional pathways in ccRCC

**Fig. 5:**
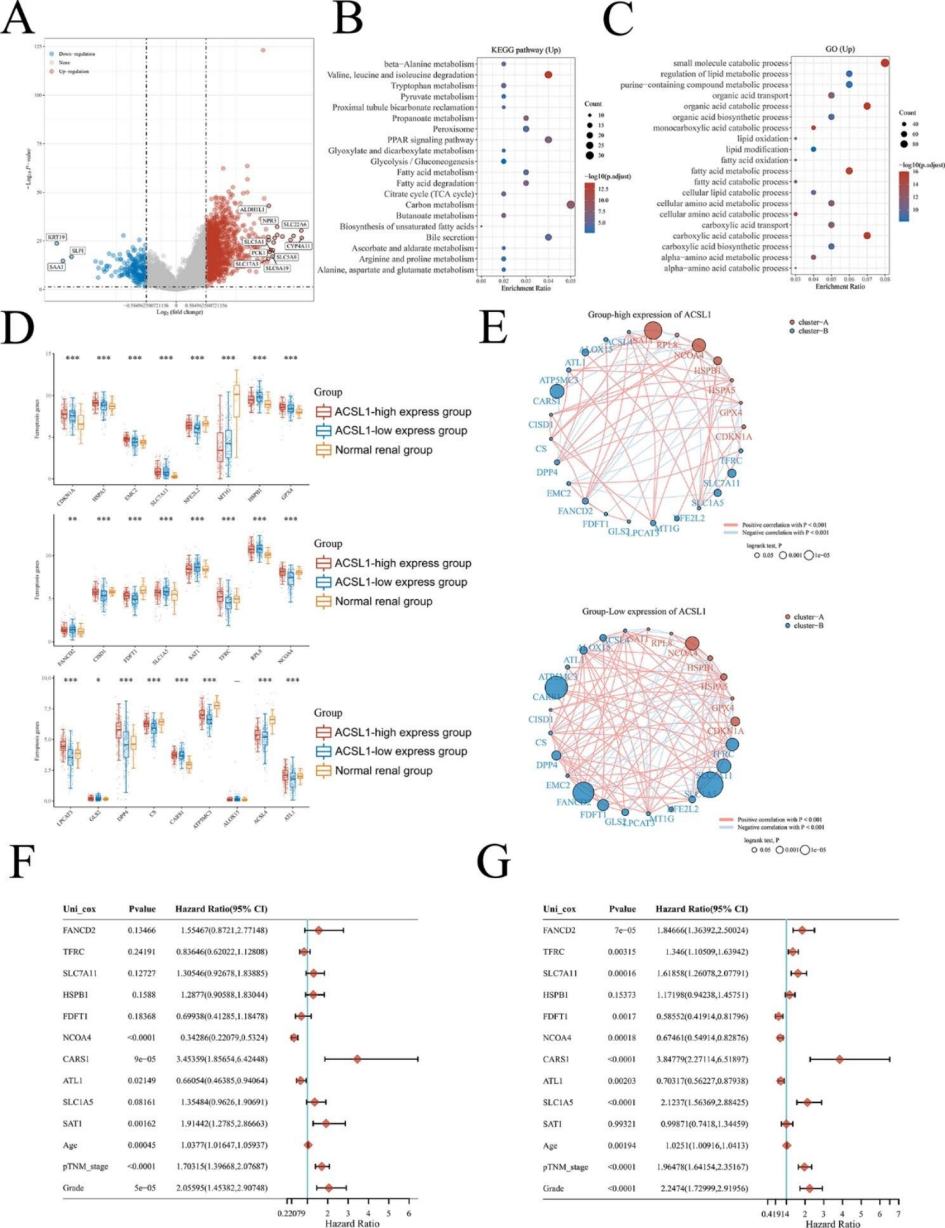
Enrichment analysis of ACSL1 high-expression group in ccRCC and the relationship of ACSL1 expression in ccRCC with the ferroptosis-related genes. **(A) The v**olcano map showed differential genes in ACSL1-high and ACSL1-low subgroups. **(B-C)** Enrichment analysis of KEGG and GO for genes up-regulated in ACSL1 high-expression group. **(D) F**erroptosis-associated genes in ACSL1 high-,low-expression groups in ccRCC and in normal renal tissues group. *P < 0.05, **P < 0.01, ***P < 0.001. **(E)** The link between ferroptosis-related genes in the ACSL1 high- and low-expression group. **(F-G)** Univariate Cox regression analysis of age, clinical stage, tumor grade and ten ferroptosis-related genes with the smallest p-value in predicting prognosis in ACSL1-high and ACSL1 low-expression group in ccRCC

Compared to the ACSL1 low-expression group, 1286 genes displayed up-regulation and 215 genes exhibited down-regulation in the ACSL1 high-expression group (Fig. 5A, P < 0.05). A full description is shown in Supplementary Table 1. By analyzing the extent of KEGG and GO enrichment, we substantiated that the up-regulated genes were principally concentrated in metabolism-associated processes and pathways, including the degradation of Valine, leucine and isoleucine, the PPAR pathway, carbon metabolism and Tryptophan metabolism. Specially, we observed a significant enrichment of fatty acid metabolism-related pathways such as fatty acid metabolism, fatty acid degradation and fatty acid metabolic processes (Fig. 5B-C). Besides, the enrichment analysis for up-regulated genes indicated that fatty acid metabolism was more activated in the ACSL1 high-expression group versus the ACSL1 low-expression group.

### High ACSL1 expression was linked to ferroptosis-related genes in ccRCC

The enrichment results highlighted that fatty acid oxidation-related signalings were predominantly involved in the ACSL1 high-expression group and that fatty acid oxidation featured prominently in regulating ferroptosis. We found that these ferroptosis related genes NFE2L2, CISD1, FDFT1, TFRC, NCOA4, LPCAT3. DPP4, CS, ATP5MC3, ACSL4 and ATL1 were obviously downregulated in ACSL1 low-expression group compared to ACSL1 high- expression group and normal kidney tissue group (Fig. 5D). These evidences suggested that the ACSL1 low-expression group had downregulated ferroptosis signal. Next, we analyzed the interaction between the 25 ferroptosis-related genes and the prognosis in ACSL1 high and low-expression groups, respectively. As presented in Fig. 5E, there was a positive correspondence between most ferroptosis-regulatory genes and prognosis in the ACSL1 low-expression group. Furthermore, we selected ten genes (FANCD2, TFRC, SLC7A11, HSPB1, FDFT1, NCOA4, CARS1, ATL1, SLC1A5 and SAT) with the smallest *P* value in predicting prognosis in the ACSL1 high-expression group and ACSL1 low-expression group, respectively, and conducted univariate Cox analysis (Fig. 5F-G). We uncovered that higher expression of NCOA4 and ATL1 was implicated in better prognosis, while CARS1 and SAT1 showed the opposite effects in the ACSL1 high-expression group. In the ACSL1 low-expression group, FDFT1, 7NCOA4, and ATL1 were related to favorable prognosis, while FANCD2, TFRC, SLC7A11, CARS1, and SLC1A5 were correlated with an inferior prognosis (Fig. 5F-G).

### Low ACSL1 expression was linked to high immunogenicity in ccRCC

**Fig. 6:**
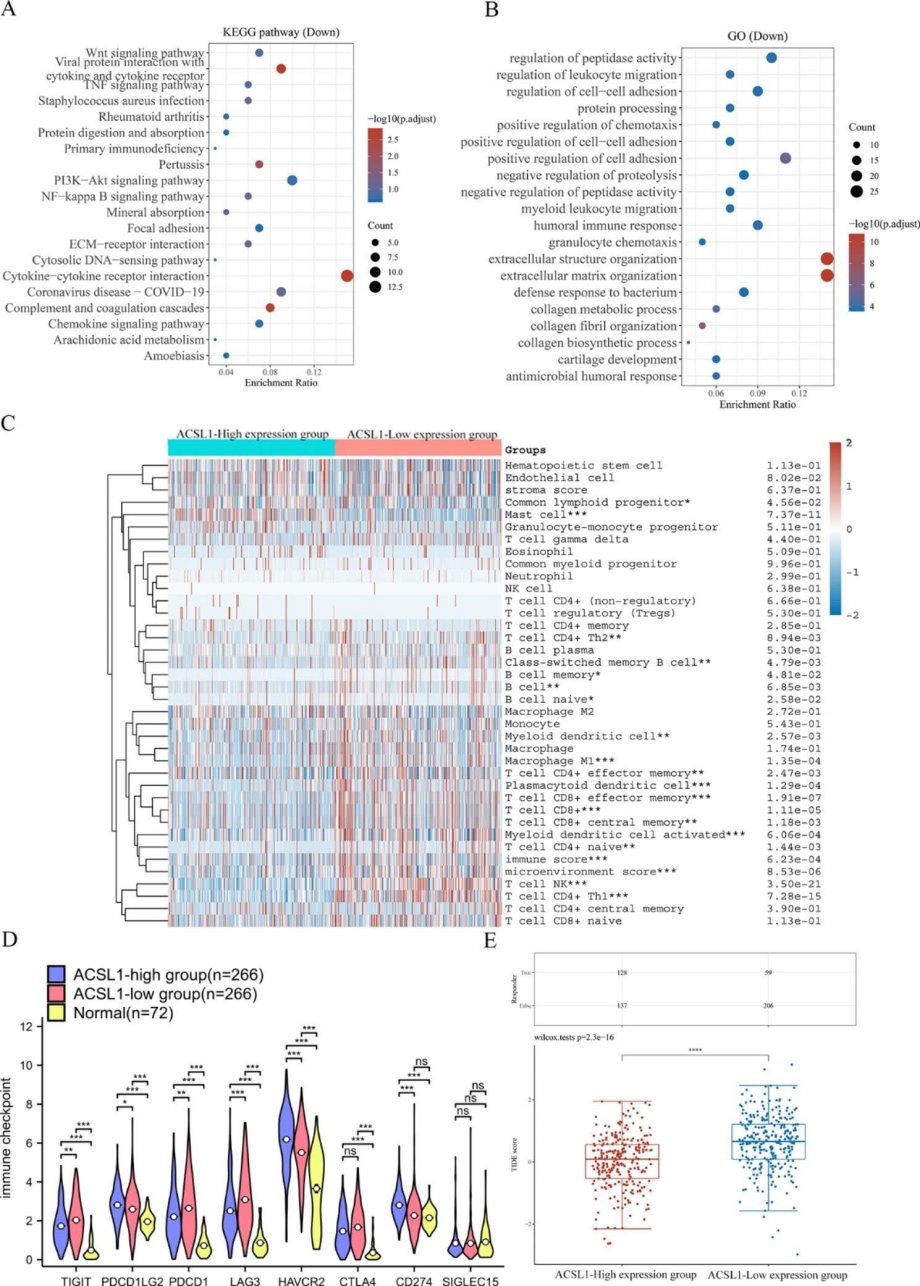
Correlations of ACSL1 low-expression with immunogenicity. **(A-B)** Enrichment analysis of KEGG and GO for genes up-regulated in the ACSL1 low-expression group. **(C)** Immune cell infiltration in ACSL1 high- and low-expression groups. *P < 0.05, **P < 0.01, ***P < 0.001. **(D)** Various expression of immune checkpoints in ACSL1 high- and low-expression groups. *P < 0.05, **P < 0.01, ***P < 0.001. **(E)** TIDE scores of ACSL1 high- and low-expression groups

KEGG and GO enrichment results also indicated that the ACSL1 low-expression group had significantly enriched immune-associated pathways, such as cytokine-cytokine receptor interaction, complement and coagulation cascades, regulation of cell-cell adhesion, T-cell activation, humoral immune response, leukocyte cell-cell adhesion, and modulation of T cell activation (Fig. 6A-B). In the following analyses, we adopted the xCell algorithm to investigate the relationship between ACSL1 expression and immune infiltrating cells in TCGA-ccRCC samples. The findings presented that the ACSL1 low-expression subgroup had a significantly higher immune score (*P* < 0.001) and microenvironment score (*P* < 0.001). Multiple immune cells were also elevated in the ACSL1 low-expression group, including macrophage M1 (*P* < 0.001), T cell CD8+ (*P* < 0.001), CD4 + effector memory T cells (*P* < 0.001), B cells (*P* < 0.001), and T cell NK (*P* < 0.001) (Fig. 6C).

Besides, the distinct expression levels of immune checkpoints among the ACSL1 low-expression group, ACSL1 high-expression group and normal renal tissues group were compared (Fig. 6D). Almost all immune checkpoints except SIGLEC15 had higher expression level in tumor tissues compared to normal kidney tissues. And higher expression of, LAG3, PDCD1 and TIGIT, and lower expression levels of CD274, HAVCR2 and PDCD1LG2 were found in the ACSL1 low-expression subgroup versus the ACSL1 high-expression subgroup.These results suggest that immune cells-expressed immune checkpoints (LAG3, PDCD1 and TIGIT) may participate in the immune escape of tumor cells in the ACSL1 low-expression subgroup of ccRCC, and ACSL1 low-expression group could be suitable for the combination of LAG3 or TIGIT with PD-1 inhibitors.

Moreover, the TIDE algorithm was applied to probe the potential ICB response between high and low expression levels of ACSL1 in the TCGA-ccRCC samples. The TIDE score was obviously reduced in the ACSL1 high-expression group versus the ACSL1 low-expression group (Wilcoxon rank-sum test, *P* = 2.3e-16; Fig. 6E). The TIDE algorithm mimics immune evasion in tumors taking advantage of T cell dysfunction and exclusionary features, and higher TIDE prediction scores correlate with poorer ICB responses. Our results substantiated that the ACSL1 high-expression group might respond better to ICB versus the ACSL1 low-expression group and was more suitable for immunotherapy.

### Potential therapeutic strategies for ACSL1 low-expression subgroup of ccRCC

**Fig. 7:**
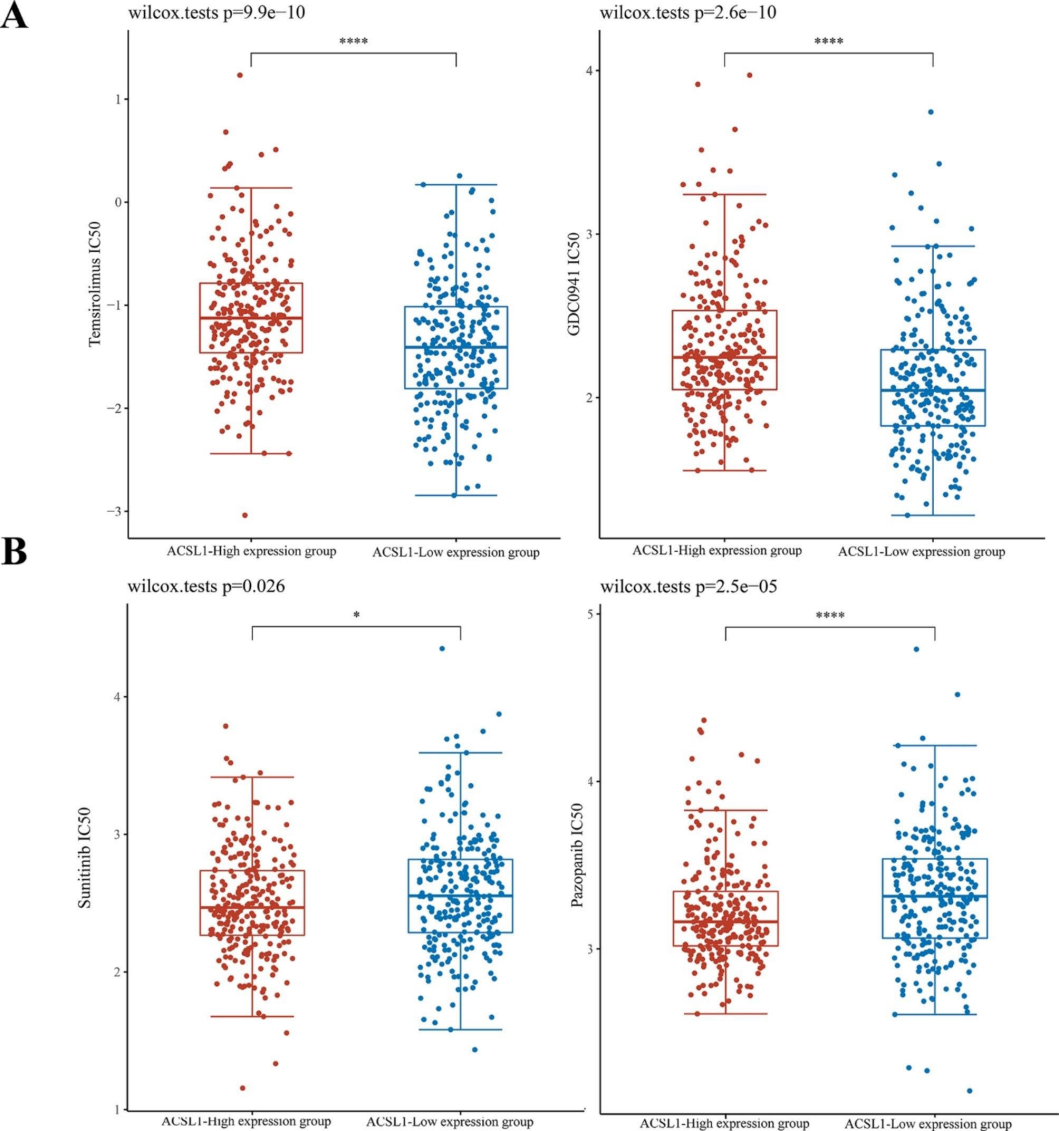
Drug sensitivities prediction based on ACSL1 expression in ccRCC. **(A)** The predicted IC_50_ values for ACSL1 high- and low-expression groups in ccRCC to Temsirolimus and Pictilisib treatment. **(B)** The predicted IC_50_ values for ACSL1 high- and low-expression groups in ccRCC to Pazopanib and Sorafenib treatment. *P < 0.05, **P < 0.01, ***P < 0.001

We found that the KEGG pathway enrichment triggered the PI3K-Akt pathway in the ACSL1 low-expression subgroup, so we evaluated the sensitivity of PI3K-Akt-mTOR pathway inhibitors, including Temsirolimus, Pictilisib (GDC0941) using data from the GSDC database. As a result, the IC50 of the ACSL1 low-expression group for Temsirolimus (*P* < 0.0001) and Pictilisib (*P* < 0.0001) was lower versus the ACSL1 high-expression group (Fig. 7A).

Considering the extensive clinical application of tyrosine kinase inhibitors (TKIs) in treating ccRCC, the sensitivity of several commonly used TKIs was also calculated. As displayed in Fig. 7B, high ACSL1 expression corroborated with the high sensitivity of sunitinib (*P* < 0.05) and Pazopanib (*P* < 0.0001). Overall, the ACSL1 low-expression subgroup of ccRCC might be suitable for PI3K pathway inhibitors, while the ACSL1 high-expression subgroup of ccRCC might be more sensitive to Sunitinib and Pazopanib.

## Discussion

In this study, we used TCGA data set to predicate the ACSL1 expression in various cancers and revealed that ACSL1 was down-regulated in 10 types of cancer, including ccRCC. On the basis of the analysis of GEO and TCGA ccRCC databases, we found the ACSL1 expression in ccRCC samples was notably lower than in normal tissues, which were validated by qRT-PCR and western blotting. Furthermore, lower ACSL1 expression was related to higher clinical stage, pathological grade and poor prognosis in ccRCC. At the same time, we evaluated the predictive role of ACSL1 expression on the prognosis of ccRCC using ROC curves and observed that ACSL1 was highly accurate in predicting the outcome of normal and tumor samples. However, many prior reports have revealed that ACSL1 was up-regulated in prostate, ovarian and colorectal cancers [12, 52, 53]. And patients with stage II colorectal cancer with higher ACSL1 levels had poorer clinical outcomes. Also, Qi L et al. [54]. disclosed that breast cancer patients with high levels of ACSL1 tended to have a poor prognosis. In conclusion, ACSL1 is potentially a prognostic and diagnostic factor for ccRCC sufferers, and the variances in ACSL1 expression between ccRCC and other tumors may account for the different tumorigenesis mechanisms of ACSL1 in diverse cancer types.

The heterogeneity of ACSL1 in various solid tumors dictates its different regulatory mechanisms. In parallel, many pathways contribute to gene dysregulation, of which epigenetic modifications are integral. Recent reports have emphasized that DNA methylation is a critical epigenetic modification of the genome that contributes to tumorigenesis [55, 56]. Modulating the DNA methylation levels of target genes can affect either oncogenes or tumor-suppressive genes, thus influencing tumorigenesis and tumor progression [57]. By analyzing the TCGA-ccRCC cohort, we disclosed that DNA methylation-related genes had a close relationship with the ACSL1 expression, so we decided to study in depth the methylation level of ACSL1. Through the SMART tool, we found a higher hypermethylation status in the promoter region of the ACSL1 gene in ccRCC tissues versus the normal tissues, particularly for the GpG island-related probes cg03498175 and cg08823975. We also observed that cg03498175, cg20823481, cg08823975, cg24277788, cg00372249, cg03977443, cg01899937 and cg111668687 had obvious negative relationship with ACSL1 expression in ccRCC tissues. More importantly, the higher extent of methylation of cg20823481, cg24277788, cg01899937 and cg111668687 led to a poorer prognosis. The findings displayed that the regions detected had a hypermethylated status and were adversely correlated with the ACSL1 expression. In brief, DNA Methylation may affect the ACSL1 expression in ccRCC. These findings inspired us to further study how methylation of different loci within ACSL1 influences the survival outcomes of ccRCC.

M6A modifications, a component of epigenetic modifications, are among the most prevalent RNA methylation modifications that affect the development and evolution of numerous tumors [58–61]. For instance, Li Y, et al. [58] revealed that RNA m6a reader YTHDF2 exhibits pronounced expression in lung adenocarcinoma tissues and induces lung adenocarcinoma cell proliferation and metastasis by targeting the AXIN1/Wnt/β-catenin pathway. Nevertheless, there were few studies about the association between m6a and ACSL1 expression in ccRCC. Here, we first uncovered that the ACSL1 expression positively correlated with the expressions of m6A writers (ZC3H13, WTAP, VIRMA, RBM15, METTL3, and METTL14), m6A readers (YTHDF1, YTHDF2, YTHDF3, YTHDC1, YTHDC2, RBMX, HNRNPC, and HNRNPA2B1), and m6A erasers (FTO and ALKBH5). More importantly, we identified that these reported m6A-related genes involved in ccRCC prognosis were grounded in ACSL1 expression. In conclusion, m6A-related genes may influence the expression profiles of ACSL1 in ccRCC samples, and the link between these m6A-related genes and prognosis is also determined by ACSL1 expression. As such, additional assays are required to confirm our predictions, with specific reference to the relationship between m6a modifications and ACSL1 expression.

ACSL1, a member of the ACSL family, is mainly responsible for the esterification of enriched LCFA (12–20 carbons) [11, 12]. For example, Yang et al. [62] have reported that down-regulating ACSL1 reduces intracellular triglyceride and cholesterol levels and hampers tumor growth. Zhang et al. [53] reported that ACSL1 induces ovarian cancer cell metastasis through regulating myristoylation and FAO. However, there was no study on the biological functions of ACSL1 in ccRCC. In our research, we substantiated that high ACSL1 expression in ccRCC is mainly associated with metabolism-related pathways, like the degradation of valine, leucine and isoleucine and the metabolism of fatty acid, carbon and tryptophan. Specially, we observed the significant enrichment of fatty acid metabolism-associated pathways like fatty acid metabolism, fatty acid degradation, and fatty acid metabolic process.

Fatty acid metabolism is notorious for triggering tumorigenesis and disease progression. Interestingly, cancer cells can reprogram the metabolic pattern of fatty acids in the TME to restrict ferroptosis and thus boost cell survival [63]. ACSL4, another member of the ACSL family, exerts a major role in the process of ferroptosis [64]. Our results demonstrated that various ferroptosis-associated genes exhibited differential expression between ACSL1 high-expression and ACSL1 low-expression subgroups, indicating the potential influence of ACSL1 in modulating ferroptosis sensitivity. However, more in-depth analysis is required to test this hypothesis. So far, anti-angiogenic targeted therapy remains one of the mainstays of treatment for patients with metastatic ccRCC. Apart from targeted therapies, the applications of mTOR inhibitors and immune checkpoint inhibitors have prolonged the survival time of ccRCC patients. Unfortunately, there are still some patients who cannot benefit from this treatment strategy. Here, we observed that several signaling pathways were differentially enriched in ACSL1-high and ACSL1 low-expression subgroups, including the PI3K-Akt pathway, immune-associated biological procedures, complement and coagulation cascades, cytokine-cytokine receptor interaction and humoral immune response. Moreover, the result of the TIDE score analysis and drug sensitivity analysis uncovered that the ACSL1 high-expression subgroup had a lower TIDE score, hinting at a superior response to ICB. In contrast, the ACSL1 low-expression subgroup had lower IC50 for Temsirolimus and Pictilisib. These results imply that ACSL1 expression could serve as a promising biomarker to guide the choice of clinical treatment plans.

Overall, we found that ACSL1 is down-regulated in ccRCC, and its expression is strongly linked to the prognosis and the clinicopathologic staging of ccRCC. We also substantiated that DNA methylation might be the main cause of ACSL1 silencing and that m6A might also impact ACSL1 expression in ccRCC. The m6A-related genes associated with prognosis were identified according to the expression levels of ACSL1 in ccRCC. High ACSL1 expression may affect tumor metabolism. In parallel, ACSL1 low-expression subgroup in ccRCC, which is marked by high immune infiltration and immune checkpoint expression, and may respond better to a combination of multiple immune checkpoint inhibitors. Meanwhile, low expression of ACSL1 in ccRCC might be suitable for PI3K pathway inhibitors, while high expression of ACSL1 might have better clinical outcomes by using TKI-targeted drugs. Overall, our discoveries suggest that ACSL1 is a prospective marker for the diagnosis, prognosis and therapy of ccRCC.

Author contributions.

Research Designer: Yang Yang and Jiayu Liang; Data analysts: Yang Yang and Jiayu Liang; Experimenters: Yang Yang, Jiayu Liang, and Junjie Zhao; Writer: Yang Yang; Instructors and reviewers of the manuscript: Qiang Wei and Zhenhua Liu. The finished manuscript has been reviewed and granted by all authors.

### Electronic supplementary material

Below is the link to the electronic supplementary material.

Supplementary Material 1

Supplementary Material 2

Supplementary Material 3

Supplementary Material 4
