# Supplementary figures and images for: The multi-omics analyses of acsl1 reveal its translational significance as a tumor microenvironmental and prognostic biomarker in clear cell renal cell carcinoma
Source: Diagn Pathol. 2023 Aug 22;18:96. doi: 10.1186/s13000-023-01384-y (PMC10463412; doi:10.1186/s13000-023-01384-y)

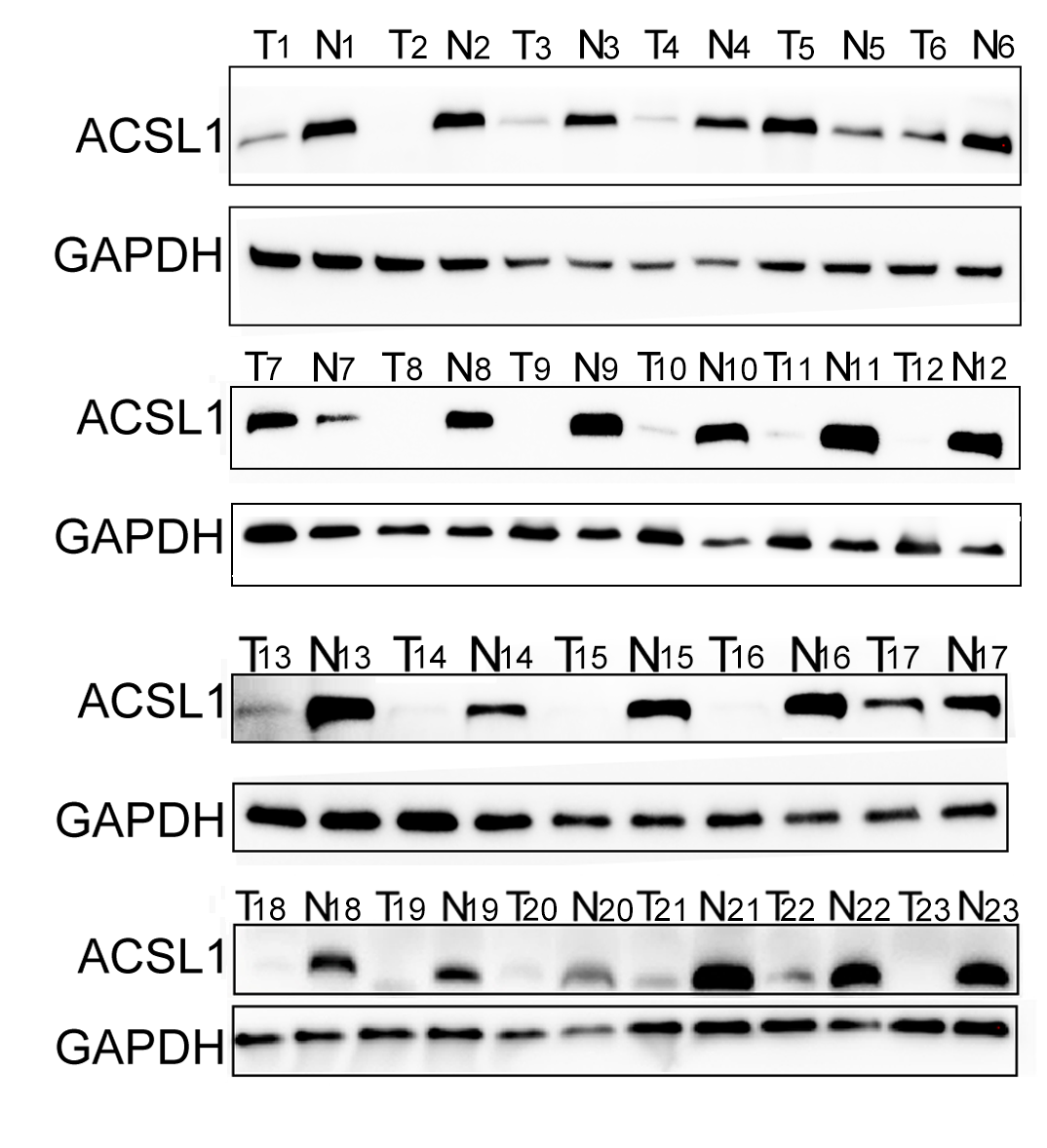

Supplement: Supplementary file 1 — Supplementary Material 1 [file 13000_2023_1384_MOESM1_ESM.png]

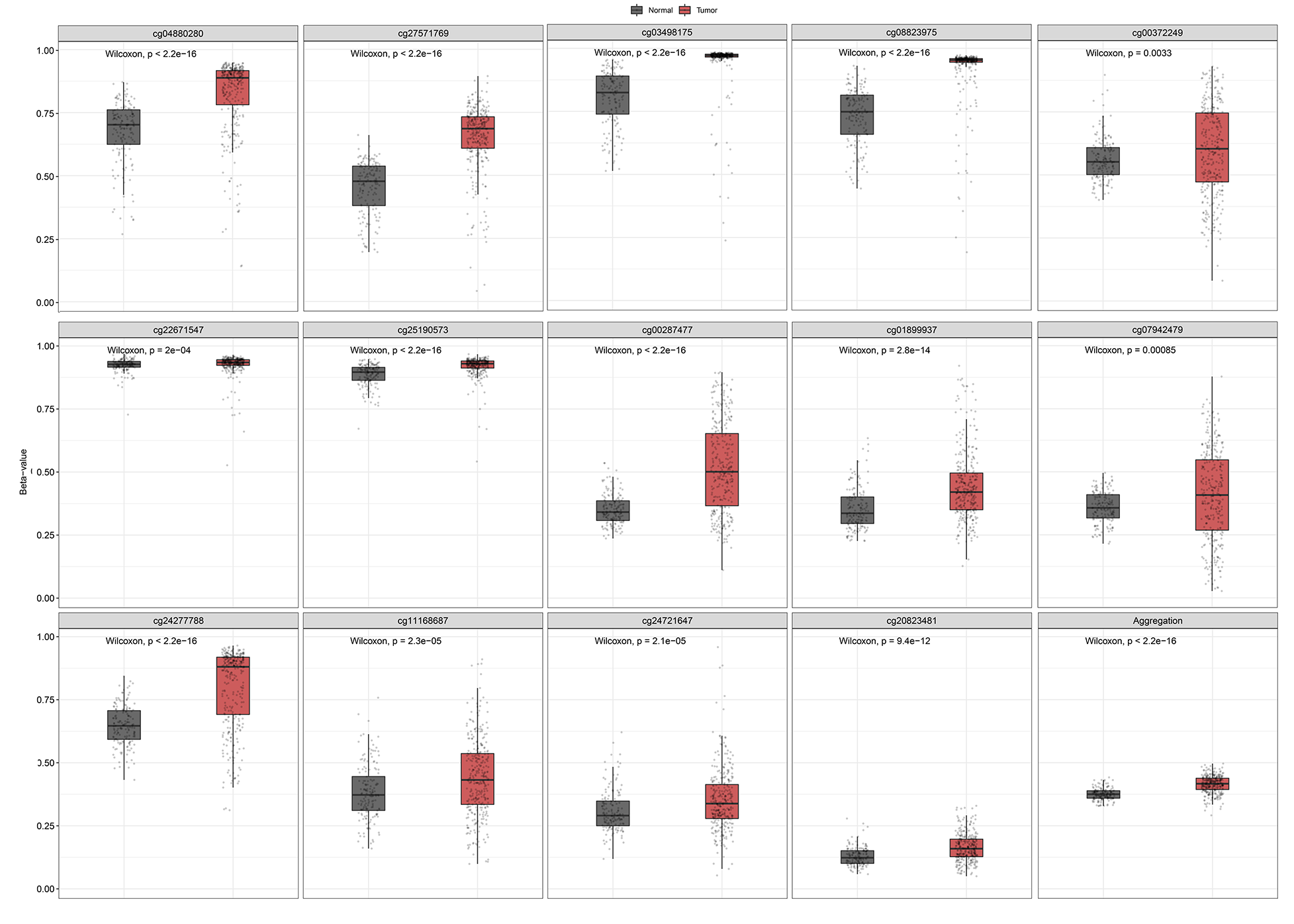

Supplement: Supplementary file 2 — Supplementary Material 2 [file 13000_2023_1384_MOESM2_ESM.png]

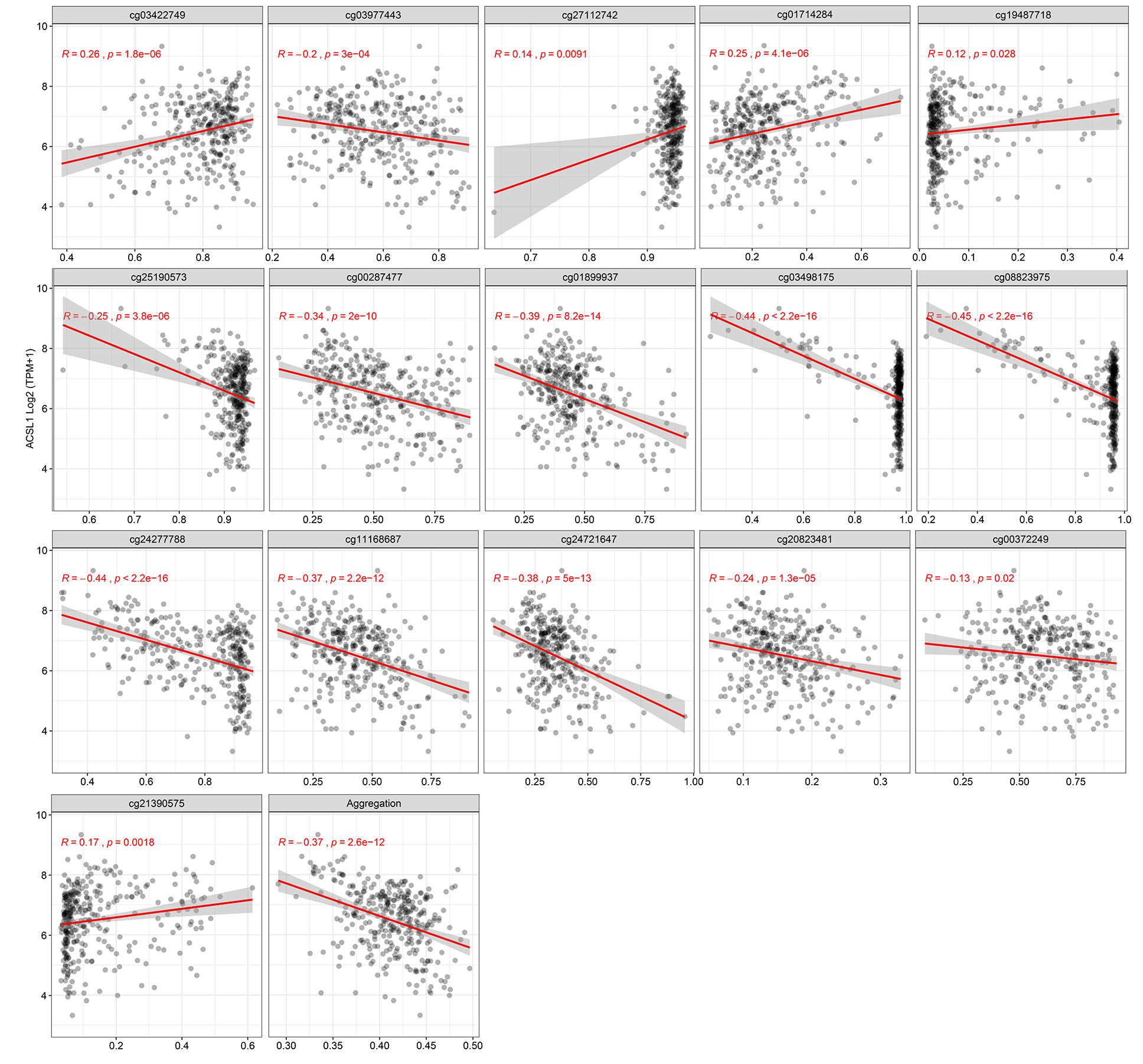

Supplement: Supplementary file 3 — Supplementary Material 3 [file 13000_2023_1384_MOESM3_ESM.png]
